# Antimicrobial resistance and natural alternatives: *in vitro* efficacy of Hungarian propolis against feline and bovine *Tritrichomonas foetus*

**DOI:** 10.3389/fvets.2025.1635358

**Published:** 2025-11-14

**Authors:** Ádám Kerek, Attila Yurt, Ábel Szabó, Barbara Tuska-Szalay, Ákos Jerzsele

**Affiliations:** 1 Department of Pharmacology and Toxicology, University of Veterinary Medicine, Budapest, Hungary; 2 National Laboratory of Infectious Animal Diseases, Antimicrobial Resistance, Veterinary Public Health and Food Chain Safety, University of Veterinary Medicine, Budapest, Hungary; 3 Department of Parasitology and Zoology, University of Veterinary Medicine, Budapest, Hungary

**Keywords:** *Tritrichomonas foetus*, propolis, antimicrobial resistance, One Health, feline trichomonosis, bovine trichomoniasis, natural antimicrobials

## Abstract

**Introduction:**

Antimicrobial resistance (AMR) is a critical One Health challenge affecting both human and animal health. *Tritrichomonas foetus*, a protozoan parasite causing reproductive and gastrointestinal disorders in cattle and cats, presents a growing threat due to limited treatment options. While nitroimidazoles such as ronidazole remain the standard of care, their use is restricted in food-producing animals and associated with emerging resistance in feline strains. Propolis, a complex natural resin produced by bees, has demonstrated antimicrobial and antiparasitic activity in other protozoan infections.

**Methods:**

This *in vitro* study assessed the minimum lethal concentrations (MLC) of ethanolic propolis tincture from the Észak-Alföld region of Hungary against feline- and bovine-derived *T. foetus* strains, compared to four nitroimidazoles.

**Results:**

Propolis showed promising activity, with an MLC of 1.25 mg/ mL for feline isolates and 0.16 mg/mL for bovine isolates after 48 h. Ronidazole demonstrated reduced efficacy against feline isolates (MLC 32 μg/mL), suggesting partial resistance, whereas bovine isolates remained susceptible (MLC 1 μg/mL).

**Discussion:**

Our findings highlight propolis as a potential alternative treatment for *T. foetus*, particularly in cattle where nitroimidazole use is prohibited. Standardizing propolis tincture and conducting *in vivo* studies will be essential to translate these results into clinical applications. This study contributes to efforts to combat AMR and develop sustainable, natural therapeutic alternatives in veterinary medicine, aligning with One Health principles.

## Graphical abstract

**Figure:**
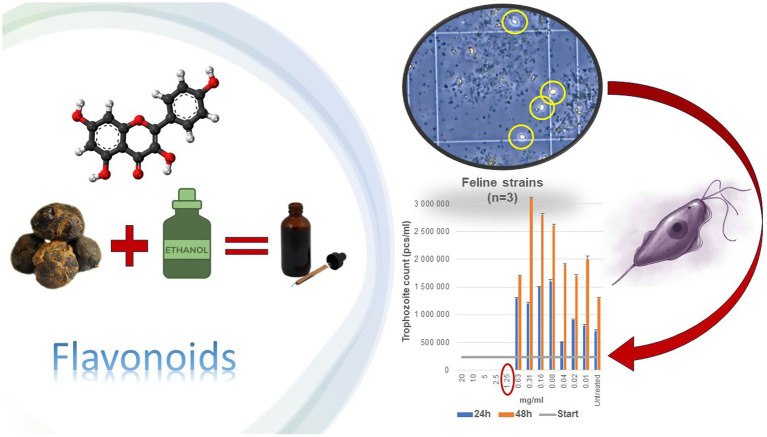
Graphical abstract illustrating the antiparasitic effects of flavonoid-rich ethanolic propolis tincture against feline Tritrichomonas foetus. The image summarizes the preparation of the hydroalcoholic propolis tincture, the presence of key flavonoids, microscopic observation of motile trophozoites, and the reduction in parasite counts following treatment at various concentrations over 24 and 48 h.

## 1 Introduction

Livestock production is the fastest-growing agricultural sector worldwide (1). However, infectious diseases remain a major challenge, causing direct losses through increased mortality and reduced productivity, pose a significant challenge (2). Furthermore, zoonotic and cross-species diseases pose risks not only to animal populations but also to human health and ecosystem integrity, as highlighted by the One Health framework (3).

Parasitic infections are particularly problematic due to their persistence and limited treatment options. Among protozoans, *Tritrichomonas foetus*, a monoflagellated parasite (4) is a significant concern, affecting both feline and bovine populations (5–7). Genetic distinctions between the feline and bovine genotypes of *T. foetus* have been identified, including single nucleotide polymorphisms (SNPs) in the internal transcribed spacer-2 (ITS-2) region and polymorphisms in elongation factor-1-alpha and cysteine protease 8 sequences (4–6, 8).

In domestic cats, *T. foetus* infections are most common in breeding facilities (9, 10), shelters (11) and exhibition settings (12, 13). Infected cats typically present with gastrointestinal signs such as diarrhea, anorexia, weight loss, abdominal pain, and chronic colitis (14, 15). Metronidazole and tinidazole show limited efficacy (16, 17) and are often unable to fully eradicate the parasite completely (18). Thus, veterinarians often turn to ronidazole (19). Ronidazole is approximately 10 times more effective than metronidazole (20), but is not authorized for cats (21). Despite high doses and prolonged use, ronidazole only achieves a 65% improvement rate against feline *T. foetus* and can lead to neurotoxic clinical signs in treated animals (14, 15, 20, 22, 23). Consequently, ronidazole is not authorized for use in cats in the European Union (24, 25).

In cattle, *T. foetus* is a sexually transmitted parasite, prevalent in regions practicing natural insemination (26). Infected bulls act as asymptomatic reservoirs (27), while infected cows may suffer from vaginitis, spontaneous abortion (28), pyometra, and other reproductive disorders, that lead to significant economic losses (26).

Although zoonotic transmission is rare, a documented case in an immunosuppressed patient underscores the potential risk (29). Due to its efficacy, safety, and excellent pharmacokinetic properties, secnidazole has been successfully used in the treatment of human *Trichomonas vaginalis* (30, 31), and giardiasis in dogs (32) and cats (33). The group of 5-nitroimidazoles remains the primary treatment option for trichomoniasis in human and veterinary medicine (34–39). However, their use in food-producing animals is prohibited (40), leaving cattle infections largely without effective treatment.

The growing antimicrobial resistance (AMR) of protozoans and the limitations of current treatments underscore the urgent need for novel therapeutic strategies (41). The desire for safer, more effective alternatives has driven interest in plant-based extractions, oils (42), antimicrobial peptides (43) and propolis (44).

Propolis is a resinous, bee-derived natural product that has garnered significant attention for its antimicrobial and antiparasitic properties. It has shown promise as a treatment for infection due to the bioactive compounds it contains. Its composition — approximately 50% resin, 30% wax, 10% essential oils, 5% pollen, and 5% organic components (45–49) — is influenced by geographical (50), botanical (45, 46, 51), and climatic factors (52), as well as by seasonal variation (45, 46), and the genetics of the bees (46, 47). Extraction methods also play a critical role in determining propolis efficacy (53). The antimicrobial efficacy of propolis is strongly correlated with its flavonoid and phenolic content, as these compounds are known to disrupt microbial metabolic pathways, inhibit enzyme activity, and induce oxidative stress in pathogens (54–56). Its anti-protozoal effects are thought to involve disruption of phospholipid metabolism, leading to cell lysis (57). Specific components such as rosmarinic acid (58), apigenin (59, 60), resveratrol (61), kaempferol (62), quercetin (63), and caffeic acid (64), can contribute to its efficacy through mechanisms such as increased reactive oxygen species (ROS) production, cytoplasmic vacuolization (65, 66), and inhibition of surface protein complexes (67, 68).

Given these promising properties, this study aimed to evaluate the *in vitro* efficacy of ethanolic propolis extracts from the Észak-Alföld region of Hungary against *T. foetus* strains isolated from cats and cattle, in comparison to established nitroimidazole treatments (metronidazole, ronidazole, tinidazole, and secnidazole). We sought to identify propolis as a potential natural alternative for managing *T. foetus* infections, particularly in light of increasing AMR and the One Health imperative for sustainable and effective therapies.

## 2 Materials and methods

### 2.1 Propolis extract preparation

The raw propolis tincture used in this study was sourced from the Northern Great Plain (Észak-Alföld) region of Hungary. The tincture was prepared by combining 1,000 *g* of propolis with 3,000 mL of 96% ethanol and 1,000 mL of glycerol. A conventional extraction method was employed, wherein the powdered crude propolis was macerated for 3 weeks at room temperature in a sealed, light-protected vessel. The undissolved components were then removed using filter paper (69). The addition of glycerol during extraction enhanced the yield of active ingredients by facilitating a more polar extraction process, as described in the literature (70). A final propolis concentration of 200 mg of raw propolis per mL of solvent, corresponding to the ratio used during tincture preparation (1,000 g propolis in 4,000 mL solvent mixture). Thus, the extract represents a hydroalcoholic propolis tincture containing glycerol as a co-solvent, in line with established preparation methods reported in the literature.

### 2.2 Parasite isolation

The feline strain of *T. foetus* was isolated from infected cats within a breeding facility in Budapest, Hungary. Samples were collected by a veterinarian for diagnostic purposes (T1–10; samples collected in November 2022) using a transport broth medium (InPouch TF-Feline, Biomed Diagnostics, White City, OR, USA). Despite previous treatment with ronidazole, chronic parasitic carriage persisted in the population. Positive samples were confirmed using polymerase chain reaction (PCR) with the QIAmp DNA Stool Mini Kit (Qiagen GmbH, Hilden, Germany). Primary feline cultures were cryopreserved in 10% DMSO in liquid nitrogen at −196 °C for further testing (71).

The bovine strain used was *T. foetus* (Riedmuller), Wenrich and Emerson, ATCC 30232 (LGC Ltd., Teddington, Middlesex, UK), a reference strain originally isolated from cattle.

### 2.3 Parasite culture

Maintenance and propagation of *T. foetus* were performed using *Trichomonas* cysteine peptone liver infusion medium (CPLM; Biolab Zrt., Budapest, Hungary). The medium was autoclaved at 121 °C for 15 min, followed by the addition of 70 mL of sterile inactivated horse serum (Biolab Zrt., Budapest, Hungary). A vial of *Trichomonas* selective supplement (Biolab Zrt., Budapest, Hungary) was then added, containing 500 mg/vial streptomycin and 80 mg/vial penicillin added to 425 mL of CPLM broth, to inhibit bacterial overgrowth that might outcompete the parasite. Fresh medium was prepared weekly and stored at 4 °C, while cultures were maintained in 15 mL centrifuge tubes at 37 °C under aerobic conditions. The cultures were passaged every 2 days to ensure their growth remained in the log phase.

Parasite morphology was observed using a Leica Microsystems Dmi1 microscope (BioMarker Kft., Gödöllő, Hungary) at 400 × magnification. Quantification of motile trophozoite forms—characterized by their bulb-like shape, undulating membrane, and jerky flagellar movement (Supplementary Video 1)—was conducted using a Bürker chamber (DIN12847, VWR International, LLC., Debrecen, Hungary) and a standard cell-counting formula.

The initial trophozoite count was determined 24 h post-incubation of the received samples. Mean cell counts were calculated across 25 large squares in the chamber. A twofold dilution was achieved by adding 20 μl of sterile isotonic saline to 20 μl of the suspension. The average cell count was then multiplied by the dilution factor and normalized using a factor of 2.5 × 10^5^, yielding the final count in pcs/mL.

### 2.4 Determination of the total phenolic content and total flavonoids content

The total phenolic content (TPC) of the propolis tincture was assessed using the Folin–Ciocalteu method (72) with gallic acid as the standard. For the analysis, 200 μg/mL of the propolis tincture was mixed with 500 μl of Folin–Ciocalteu reagent (10% v/v) and 500 μl of sodium carbonate (2% w/v). The reaction mixture was incubated at room temperature, without light, for 1 h. Absorbance was measured at 700 nm using a Hach DR6000 spectrophotometer (Hach Lange Kft., Budapest, Hungary), with a blank (reaction mixture without propolis tincture) serving as the control. A calibration curve was constructed with standard solutions of gallic acid (0.01–0.5 mM; Merck Life Science Ltd., Budapest, Hungary). The resulting regression equation was y = 0.0061x + 0.0278, with R^2^ = 0.9987. The TPC results were expressed as gallic acid equivalent (GAE) in milligrams (mg) per gram (g) of the dry weight (DW) of the propolis tincture.

The total flavonoid content (TFC) was determined using the aluminum chloride colorimetric method as described by Dias et al. (73). In this analysis, 125 μl of 1 mg/mL propolis tincture was mixed with 625 μl of distilled water and 37 μl of 5% sodium nitrite solution. After 5 min, 75 μl of 10% aluminum chloride solution was added, followed by 250 μl of 1 M sodium hydroxide and 137 μl of distilled water. The mixture was vortexed thoroughly, and the intensity of the resulting pink coloration was measured at 510 nm using a Hach DR6000 spectrophotometer, with a blank as the control. A calibration curve was constructed with standard solutions of catechol (0.022–1.5 mM; Merck Life Science Ltd., Budapest, Hungary). The resulting regression equation was y = 0.0049x + 0.0152, with R^2^ = 0.9979. The TFC results were expressed as catechol equivalent (CAE) in mg per g of DW of propolis.

Both TPC and TFC determinations were conducted in triplicate to ensure accuracy and reliability.

### 2.5 LC–MS/MS profiling of selected bioactive compounds

To confirm the accuracy of the colorimetric determinations of total phenolic and flavonoid content, the propolis tincture was additionally analyzed by LC–MS/MS using a SCIEX Exion LC 2.0 UHPLC system (AB Sciex LLC, Framingham, MA, USA) coupled to a SCIEX QTRAP 4500 triple quadrupole mass spectrometer (AB Sciex Pte. Ltd., Singapore) under identical chromatographic conditions. In parallel with the colorimetric assays, the concentrations of three major phenolic constituents of propolis, caffeic acid phenethyl ester (CAPE), pinocembrin, and galangin were quantified based on literature reports of their antimicrobial relevance (74–77). Separation was achieved on a Merck Purospher STAR RP-18 column (150 × 4.6 mm, 3 μm particle size; Merck KGaA, Darmstadt, Germany) maintained at 30 °C. The mobile phases consisted of solvent A (water with 0.1% formic acid) and solvent B (acetonitrile with 0.1% formic acid), with a gradient elution from 20 to 80% B over 20 min. The injection volume was 10 μl and the flow rate was 0.8 mL/min. Multiple reaction monitoring (MRM) mode was applied, with compound-specific precursor–product ion transitions optimized for each analyte.

### 2.6 The propolis and nitroimidazole treatment

Parasite growth and viability were assessed following previously described methodologies (78, 79). During the treatment phase, a hydroalcoholic propolis tincture (200 mg/mL in 96% ethanol with glycerol) was employed alongside a control sample. A two-fold dilution series was generated to determine the lowest concentration where the parasites die out. Unlike standard turbidity assessments, this study focused on the change in trophozoite counts across dilutions. A 24-well cell culture plate (VWR International, LLC., Debrecen, Hungary) was utilized. The potential effects of the solvent (ethanol) were also evaluated independently to distinguish the impact of the active compounds in the propolis tincture from that of the solvent.

Each well was filled with 3 mL of CPLM broth, except for the first column. The propolis tincture stock solution (200 mg/mL, well A1) and 96% ethanol-only control (well C1) underwent an initial tenfold dilution (0.3 mL + 2.7 mL), followed by sequential two-fold dilutions across the wells (Table 1). The dilution series for the propolis tincture and ethanol solvent ensured that any observed effects were attributable to propolis’ active compounds rather than the solvent.

**Table 1:** Dilution series of starting propolis tincture and ethanol solvent.

Dilution series of 200 mg/mL propolis tincture						
Well	A1	A2	A3	A4	A5	A6
Dilution	10×	20×	40×	80×	160×	320×
mg/mL	20	10	5	2.5	1.25	0.62
Well	B1	B2	B3	B4	B5	B6
Dilution	640×	1,280×	2,560×	5,120×	10,240×	20,480×
mg/mL	0.31	0.16	0.08	0.04	0.02	0.01

Dilution series of 96% ethanol solvent						
Well	C1	C2	C3	C4	C5	C6
Dilution	10×	20×	40×	80×	160×	320×
%	9.6	4.8	2.4	1.2	0.6	0.3
Well	D1	D2	D3	D4	D5	D6
Dilution	640×	1,280×	2,560×	5,120×	10,240×	20,480×
%	0.15	0.07	0.04	0.02	0.01	0.005

The upper two rows of the plate contained the extracted propolis dilution along with its solvent, while the lower two rows served as controls, containing only the solvent diluted to the same concentrations as those in the upper rows.

The initial parasite suspension was prepared at approximately 2.4 × 10^5^ cells/mL for the feline strain and 5 × 10^4^ cells/mL for the bovine strain, based on proliferation rates consistent with the literature (80–84). Each well was inoculated with 50 μl of the suspension.

Nitroimidazole derivatives stock solutions (ronidazole, metronidazole, tinidazole, and secnidazole) were prepared (Merck KGaA, Darmstadt, Germany) at 1024 μg/mL in DMSO and distilled water (85). A two-fold dilution series was created in CPLM broth across the first two rows of a 24-well plate, with the solvent undergoing an equivalent dilution series. Positive (containing parasites without active compounds) and negative (lacking parasites and active compounds) control plates were included alongside treatment groups. Cultures were incubated at 37 °C under aerobic conditions.

Parasite counts were performed at 24 h and 48 h using a Bürker chamber. Minimum lethal concentrations (MLC) was defined as the lowest concentration at which no motile organisms were observed (79).

Statistical analysis of trophozoite count variations was conducted using the Kruskal-Wallis test in R version 4.3.0 (86, 87). Treatment effects were analyzed across species, strains, treatment durations, and concentrations.

## 3 Results

### 3.1 Total phenolic and flavonoid content determination

In Hungary, the primary botanical sources of propolis are poplar (*Populus* spp.) and birch (*Betula* spp.) buds (47). These sources significantly influence the concentration of phenolic and flavonoid compounds. The total phenolic content (TPC) and total flavonoid content (TFC) were measured dried extract obtained by evaporating the hydroalcoholic propolis tincture. The TPC was determined to be 37.9 ± 0.08 mg gallic acid equivalent (GAE)/g of dried extract, while the TFC was 19.2 ± 0.05 mg catechol equivalent (CAE)/g of dried extract.

**Figure 1:**
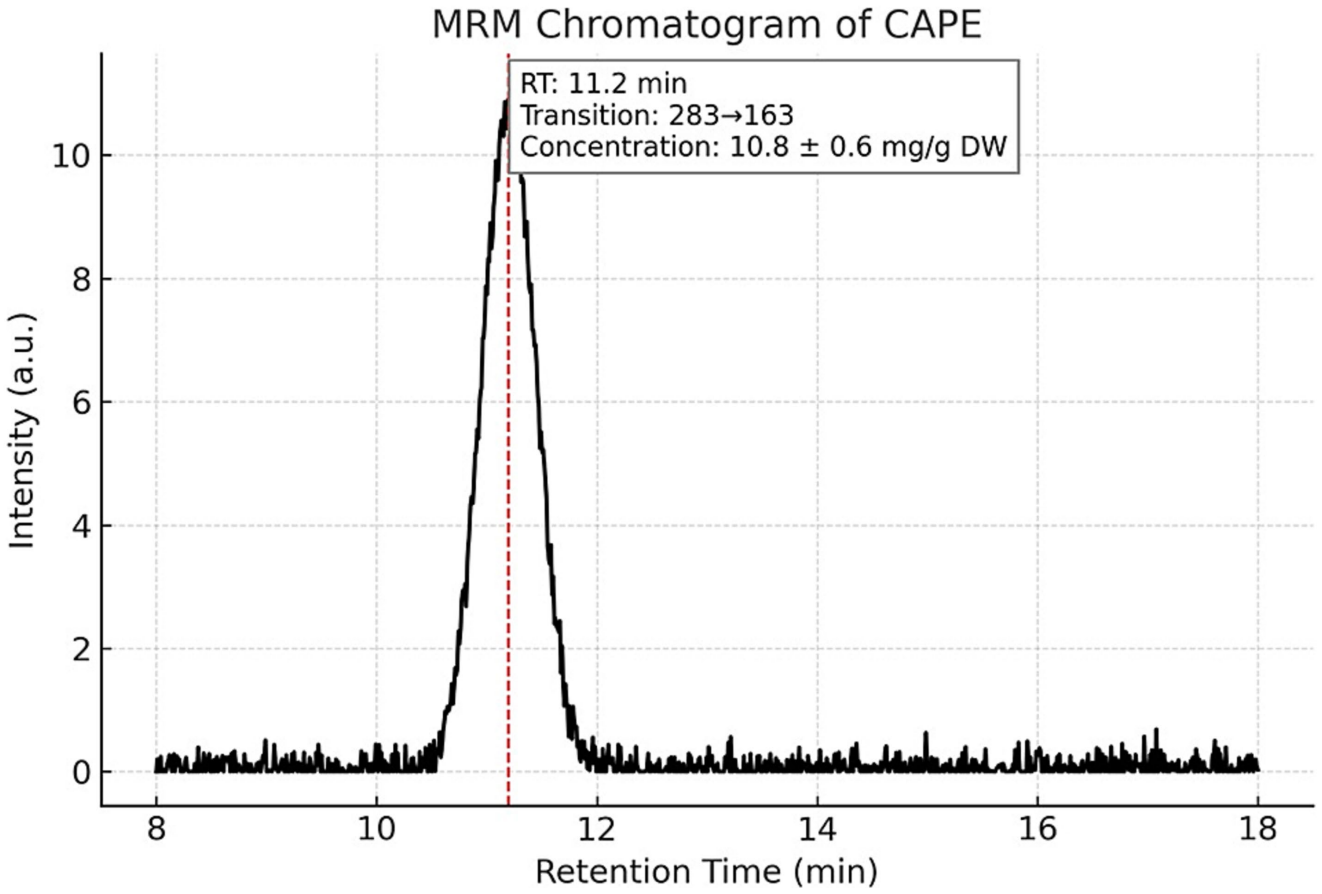
Multiple reaction monitoring (MRM) chromatogram of caffeic acid phenethyl ester (CAPE) in the propolis tincture. Representative MRM chromatogram of caffeic acid phenethyl ester (CAPE) detected by LC–MS/MS in the ethanolic propolis tincture. The compound was identified using a specific transition with *m/z* 283 and eluted at a retention time (RT) of 11.2 min. CAPE concentration was quantified at 10.8 ± 0.6 mg/g dried extract. Chromatographic separation was achieved under gradient conditions using a Merck Purospher STAR RP-18 column (150 × 4.6 mm, 3 μm) at 30 °C.

**Figure 2:**
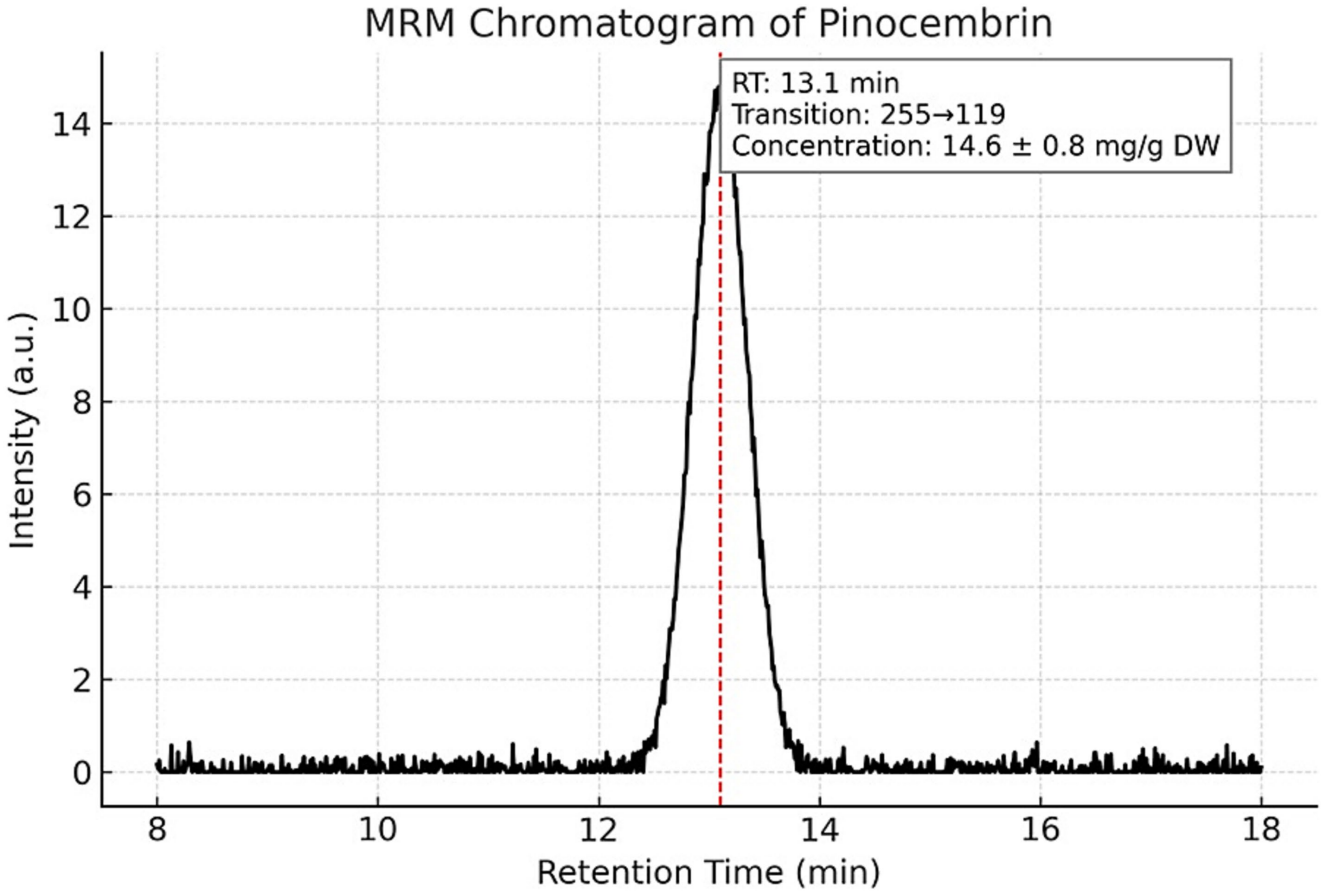
Multiple reaction monitoring (MRM) chromatogram of pinocembrin in the propolis tincture. LC–MS/MS MRM detection of pinocembrin (*m/z* 255) in the analyzed propolis tincture. The compound eluted at 13.1 min and was quantified at 14.6 ± 0.8 mg/g dried extract. The chromatographic method and instrumental parameters were identical to those described for CAPE. The distinct peak confirms the presence and retention behavior of pinocembrin among the main phenolic constituents.

**Figure 3:**
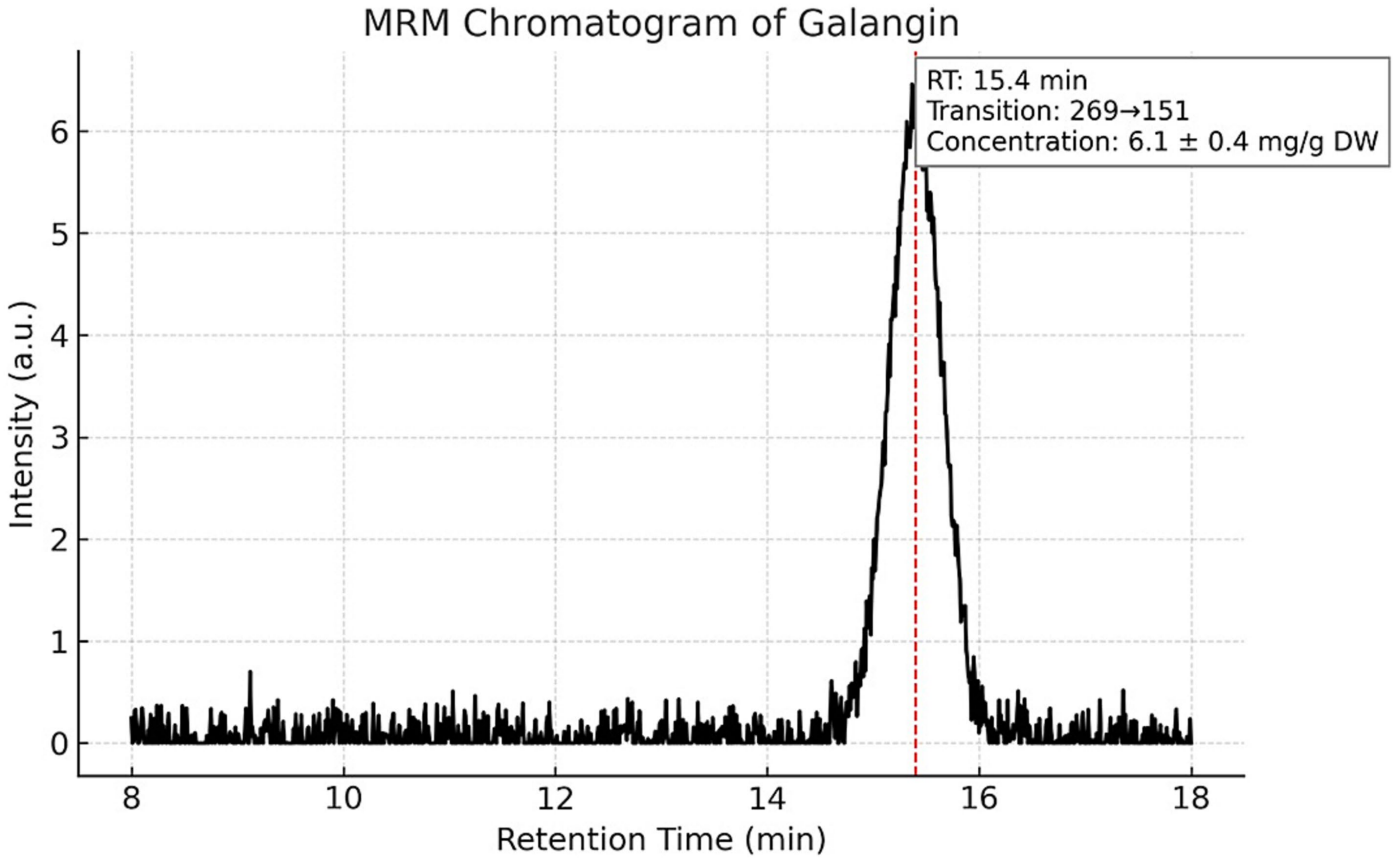
Multiple reaction monitoring (MRM) chromatogram of galangin in the propolis tincture. Chromatographic profile of galangin (*m/z* 269) obtained by LC–MS/MS in MRM mode. The analyte eluted at a retention time of 15.4 min and was present at a concentration of 6.1 ± 0.4 mg/g dried extract. This compound, together with CAPE and pinocembrin, accounts for 83.1% of the total phenolic content (TPC), indicating its strong contribution to the bioactive profile of the propolis tincture.

Targeted LC–MS/MS quantification of three phenolic constituents with well-documented antimicrobial relevance revealed concentrations of 10.8 ± 0.6 mg/g dried extract for CAPE (Figure 1), 14.6 ± 0.8 mg/g DW for pinocembrin (Figure 2), and 6.1 ± 0.4 mg/g DW for galangin (Figure 3). These compounds together account for 83.1% of the measured TPC, aligning well with values reported for temperate zone propolis. This profile underscores the substantial contribution of these constituents to the tincture’s overall bioactivity.

**Figure 4:**
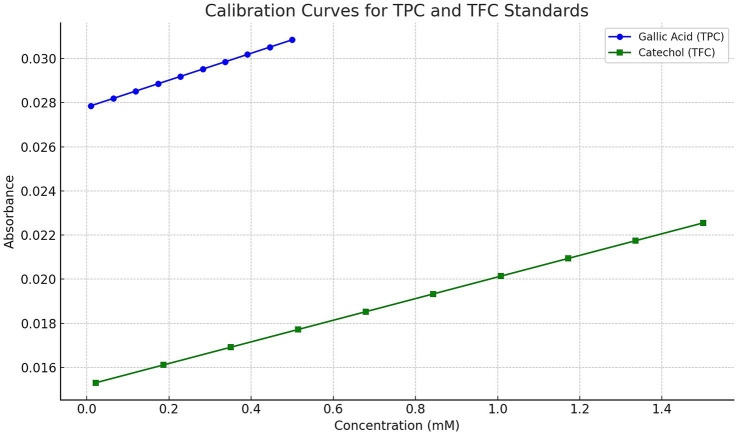
Calibration curves for gallic acid and catechol standards used in the determination of total phenolic content (TPC) and total flavonoid content (TFC), respectively. The gallic acid standard curve was constructed over the range of 0.01–0.5 mM, yielding the regression equation y = 0.0061x + 0.0278 with R^2^ = 0.9987. The catechol standard curve was generated over 0.022–1.5 mM, with the regression equation y = 0.0049x + 0.0152 and R^2^ = 0.9979. Absorbance measurements were taken at 700 nm (TPC) and 510 nm (TFC), respectively.

Calibration curves for gallic acid and catechol standards used in the TPC and TFC assays are shown in Figure 4.

### 3.2 Viability and reproduction

**Figure 5:**
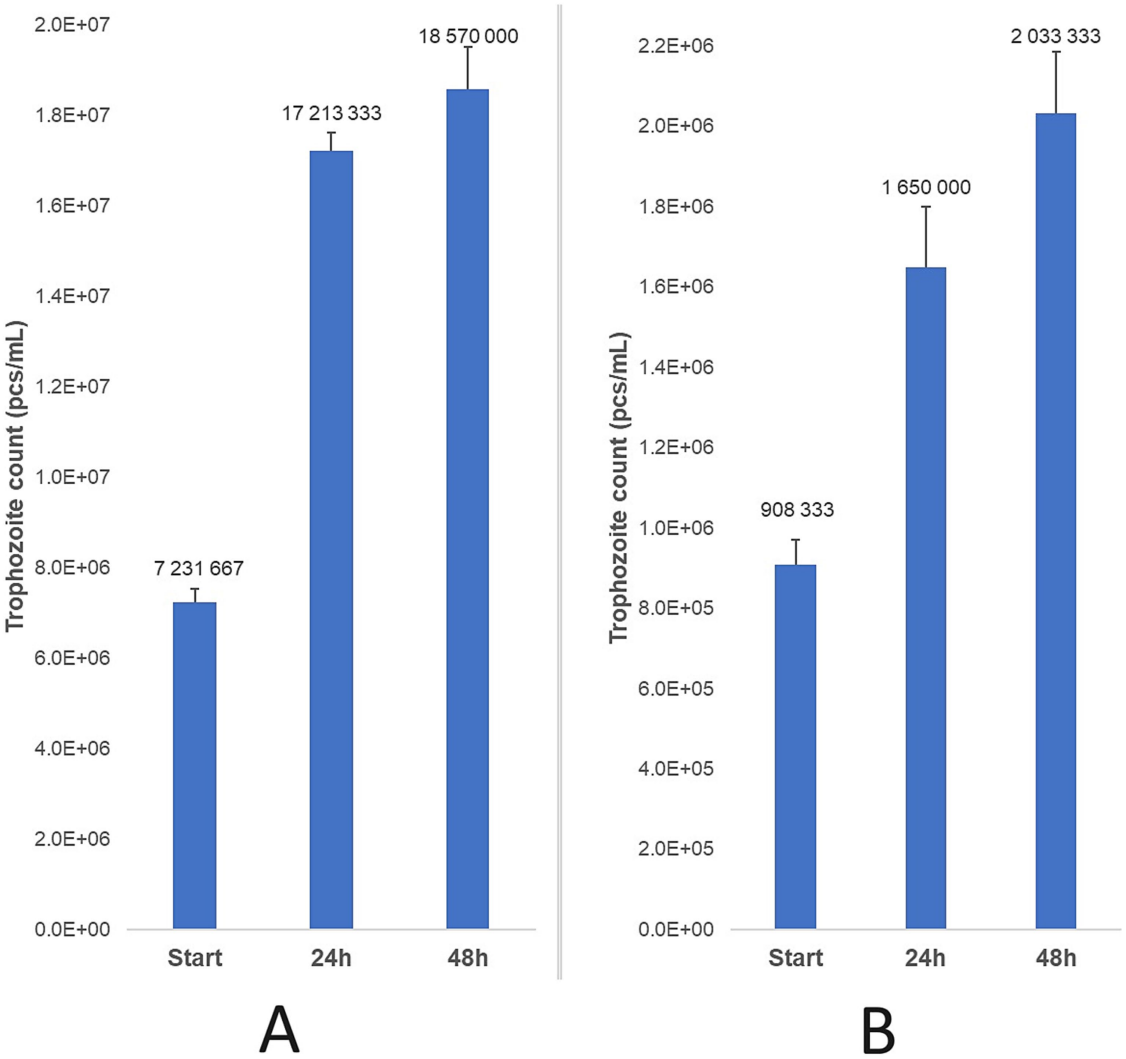
Trophozoite count progression was observed after 24 and 48 h of incubation at 37 °C for feline **(A)** and bovine **(B)** strains, relative to the initial parasite count. Data are presented as mean ± standard deviation (*n* = 3).

Parasite counts were assessed 24 and 48 h after collection, with three parallel treatments performed for each time point. For the feline strain, a marked increase in trophozoite counts was observed after 24 h (138%), followed by a smaller increase by 48 h (8%), culminating in a total increase of 157%. The bovine strain exhibited a similar, but less dramatic rise in trophozoite counts, with an 82% increase in 24 h and an additional 23% increase by 48 h, resulting in a total increase of 124% (Figure 5).

### 3.3 Propolis treatment efficacy

**Figure 6:**
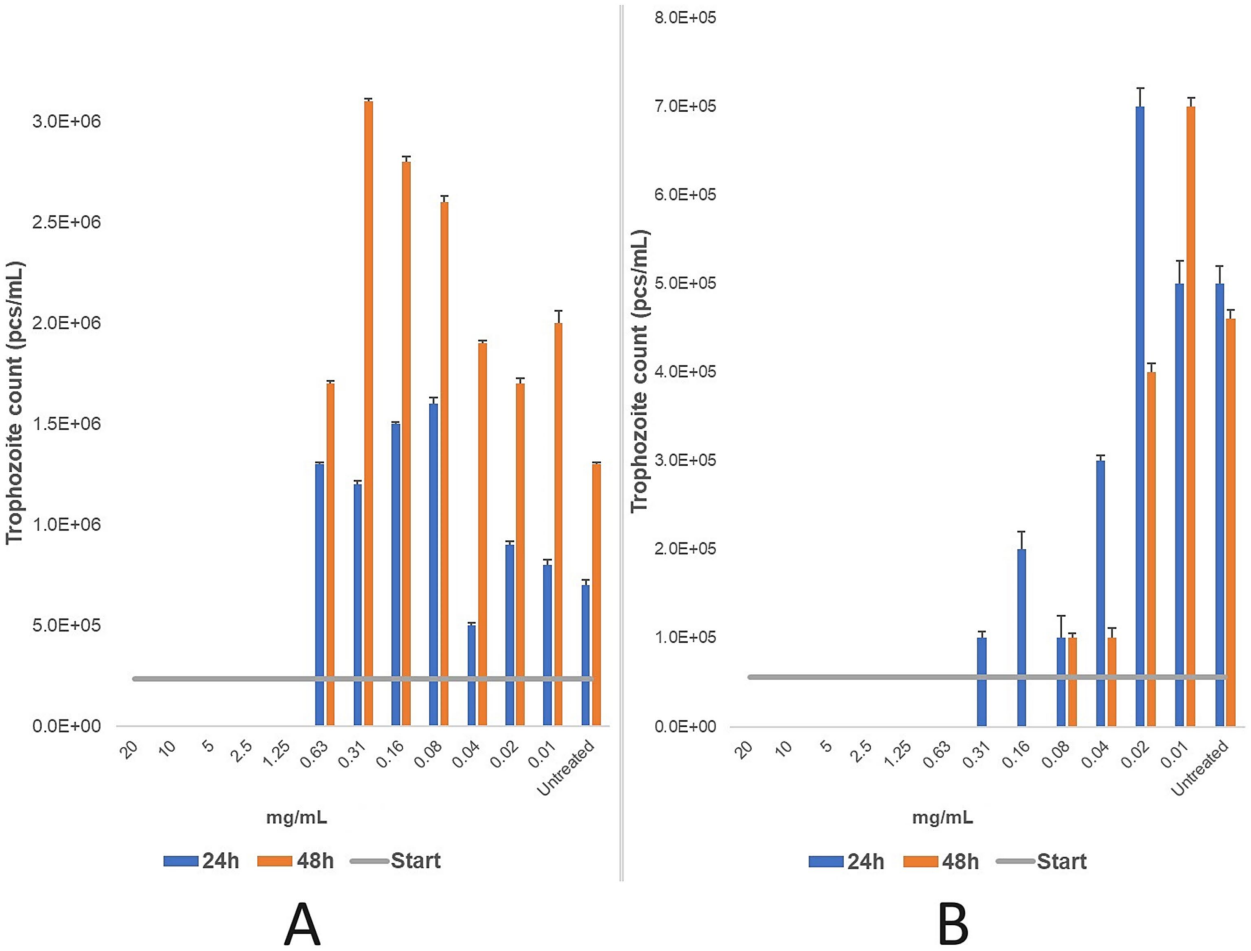
Efficacy (mg/mL) of a two-fold dilution series of propolis tincture was evaluated after 24 and 48 h of treatment for feline **(A)** and bovine **(B)** strains. The minimum lethal concentration (MLC) was determined microscopically after 24 and 48 h of incubation at 37 °C. Data are presented as mean ± standard deviation (*n* = 3). For the feline strain **(A)**, the MLC was determined to be 1.25 mg/mL. Further dilutions beyond this point showed increased in the number of parasites, indicating release from the inhibitory effects of the propolis. In the bovine strain **(B)**, the MLC was 0.63 mg/mL after 24 h of treatment, with reduced parasite counts observed at concentrations as low as 0.08 mg/mL. After 48 h of treatment, the MLC decreased to 0.16 mg/mL, with a significant antiparasitic effect persisting down to 0.04 mg/mL. Beyond this dilution, parasites were progressively released from inhibition.

Three primary *T. foetus* cultures from feline origin and three from bovine origin were tested in parallel. For the feline strain, the MLC of propolis was 1.25 mg/mL after 24 and 48 h, corresponding to a 160 × dilution of the initial propolis tincture. For the bovine strain, the MLC was 0.63 mg/mL after 24 h and decreased to 0.16 mg/mL after 48 h, representing 320 × and 1,280 × dilutions, respectively (Figure 6).

The results indicate that the feline strain was more tolerant to ethanol (9.6%), while the bovine strain exhibited greater sensitivity (4.8%). Treatment duration influenced efficacy in the feline strain, while concentration was the key determinant for the bovine strain. Statistical analysis (*p*-values) is detailed in Supplementary Table 1.

### 3.4 Nitroimidazole treatments results

Nitroimidazole derivative stock solutions (ronidazole, metronidazole, tinidazole, secnidazole) were prepared with dimethyl sulfoxide (DMSO) and distilled water. DMSO exhibited parasiticidal effects up to 1%, but below this threshold, parasites were released from inhibition. Thus, the observed effects were attributed to the active substances.

**Figure 7:**
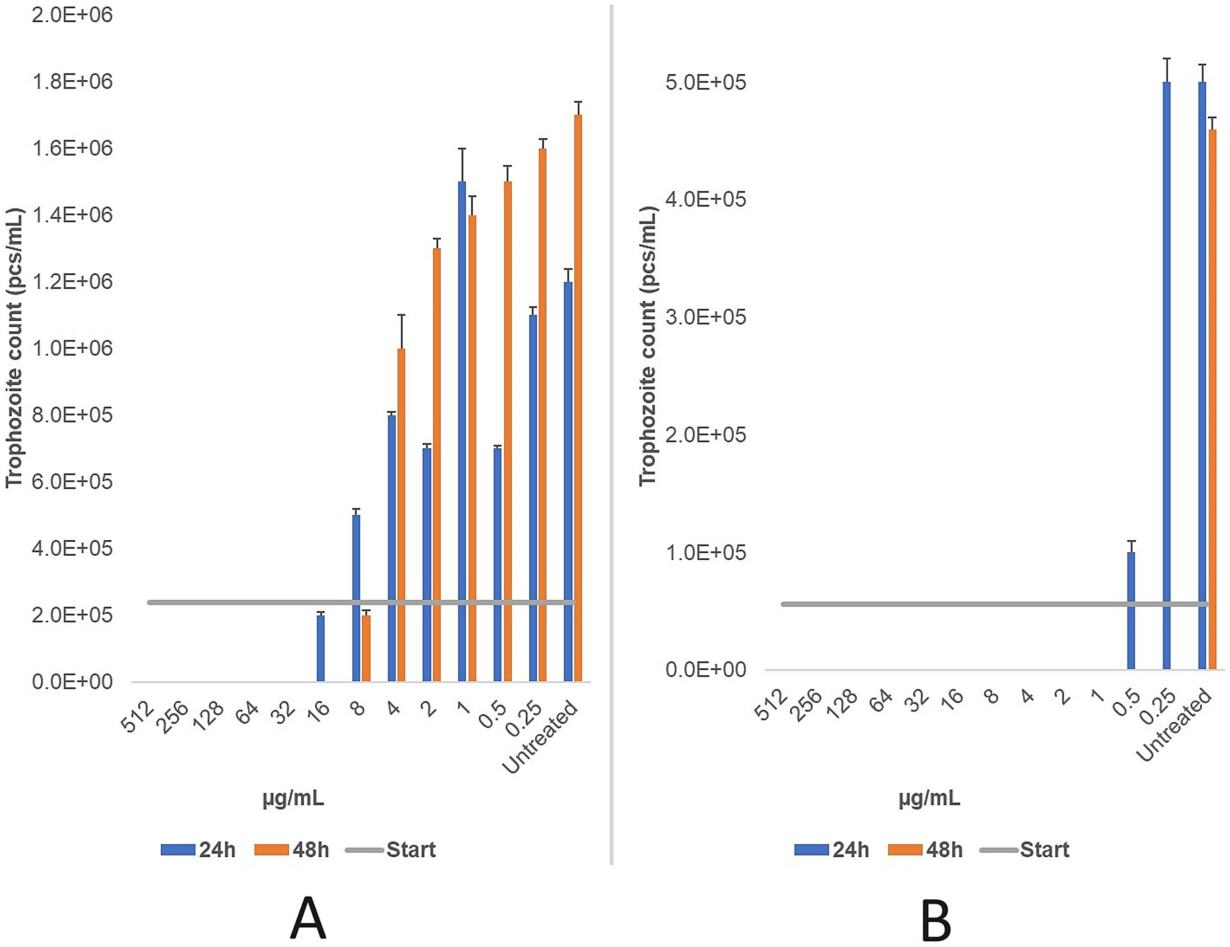
The changes in trophozoite counts were assessed after 24 and 48 h of treatment with a two-fold dilution series (μg/mL) of ronidazole for feline **(A)** and bovine **(B)** strains. The results indicate that ronidazole demonstrated the highest efficacy in the bovine strain, with a minimum lethal concentration (MLC) of 1 μg/mL, compared to 32 μg/mL in the feline strain. MLC was determined microscopically following 24 and 48 h of incubation at 37 °C. Data are presented as mean ± standard deviation (*n* = 3).

For the feline strain, the MLC of ronidazole was 32 μg/mL after 24 h and 16 μg/mL after 48 h. For the bovine strain, the MLC was 1 μg/mL after 24 h and <0.25 μg/mL after 48 h (Figure 7).

**Figure 8:**
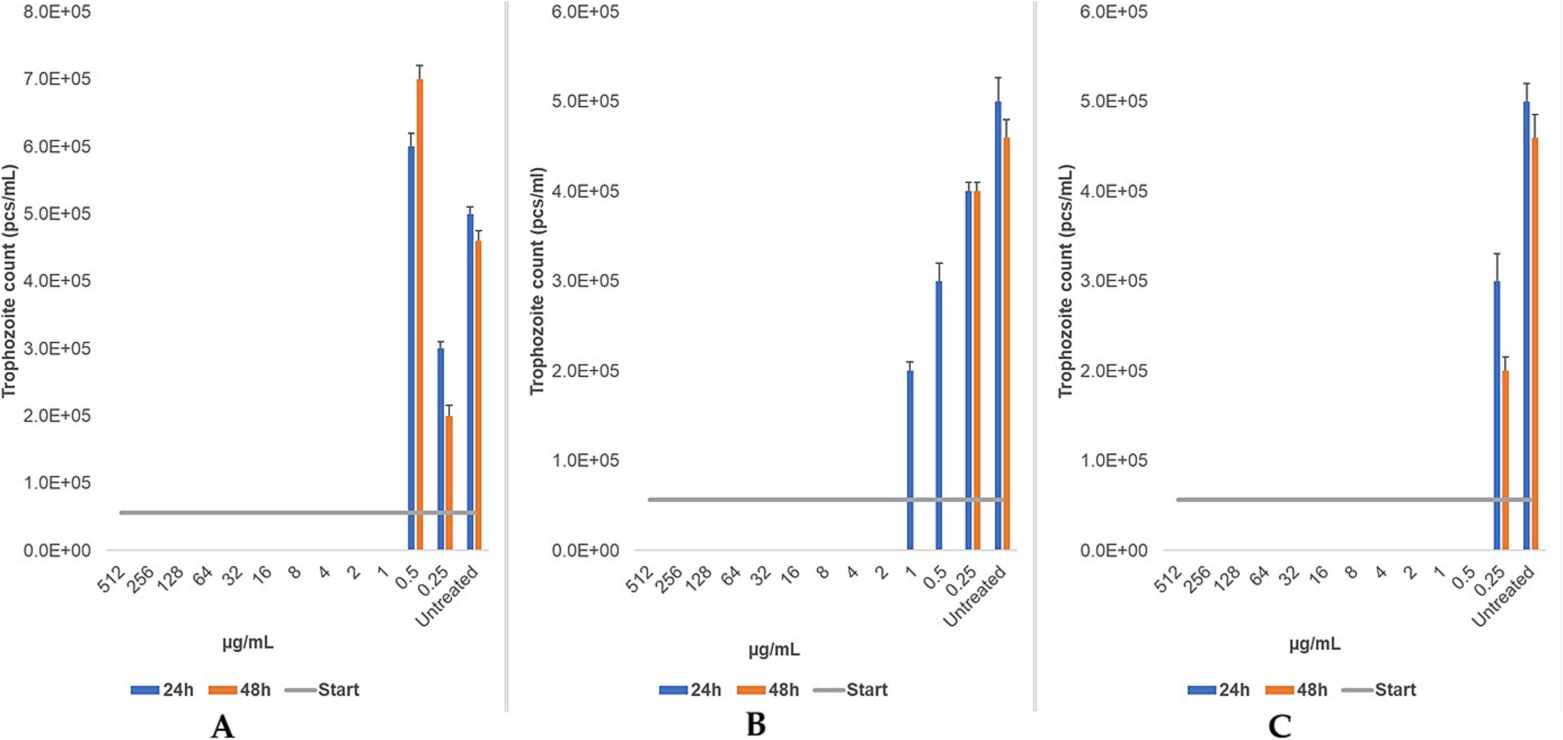
The changes in trophozoite counts after 24 h and 48 h of treatment with a two-based dilution series (μg/mL) of metronidazole **(A)**, tinidazole **(B)**, and secnidazole **(C)** are depicted for bovine strains. The results are presented as the mean and standard deviation (*n* = 3). The MLC was evaluated microscopically after 24 h and 48 h of incubation at 37 °C.

Metronidazole, tinidazole, and secnidazole were tested only on bovine strain. Metronidazole exhibited an MLC of 1 μg/mL at both 24 and 48 h. Similarly, secnidazole demonstrated a consistent MLC of 0.5 μg/mL across both time points Tinidazole’s MLC decreased from 2 μg/mL at 24 h to 0.5 μg/mL at 48 h (Figure 8). The findings demonstrate that secnidazole had the highest efficacy among nitroimidazoles, with a consistent MLC of 0.5 μg/mL, while tinidazole’s efficacy improved with longer treatment duration.

## 4 Discussion

This study evaluated the *in vitro* efficacy of a naturally derived propolis tincture and various nitroimidazole compounds against *T. foetus* isolates of feline and bovine origin. Our findings reveal clear differences in susceptibility between the two host-adapted genotypes and highlight the potential of propolis as a natural therapeutic alternative — particularly in the context of AMR and One Health priorities.

CAPE, pinocembrin, and galangin are among the most widely studied phenolic constituents of propolis, known for their potent antimicrobial and anti-inflammatory properties. Their quantification in this study provides additional chemical context for interpreting the observed biological activities. However, propolis is a complex natural product containing over 300 identified compounds, including other flavonoids, phenolic acids, terpenes, and aromatic esters (65, 77). Consequently, while the present targeted profiling captures key bioactive markers, it does not encompass the full chemical diversity of the tincture. Future studies should employ comprehensive untargeted metabolomics to expand the phytochemical coverage and investigate potential synergistic effects among constituents.

The propolis tincture achieved complete eradication of feline-origin *T. foetus* at a concentration of 1,250 μg/mL. Remarkably, bovine-origin strains demonstrated even higher sensitivity, with effective concentrations dropping to 630 μg/mL after 24 h and further to 160 μg/mL after 48 h. These results align with previous research on the anti-protozoal effects of propolis, though observed variations are likely due to differences in propolis composition, which can be influenced by geographical, botanical, and climatic factors. These findings underscore the need for standardization of propolis tincture — particularly their phenolic and flavonoid profiles — to ensure consistent efficacy and reproducibility in therapeutic applications.

Our results demonstrating the *in vitro* efficacy of Hungarian propolis against *T. foetus* are consistent with findings in other protozoan infections. For instance, Freitas et al. reported significant inhibitory effects of propolis on *Giardia lamblia* trophozoites, with complete eradication at concentrations as low as 500 μg/mL (88). Similarly, Pontin et al. observed substantial antiparasitic activity of Brazilian propolis against *Leishmania amazonensis*, suggesting a broad-spectrum potential for propolis in protozoan infections (89). These parallels reinforce the relevance of our findings and highlight propolis as a promising natural alternative for managing protozoan pathogens, particularly in the context of emerging antimicrobial resistance.

Nitroimidazoles remain the mainstay of *T. foetus* treatment, with ronidazole widely used in feline infections. In this study, ronidazole demonstrated an MLC of 32 μg/mL against feline isolates after 24 h, decreasing to 16 μg/mL after 48 h. Given that the resistance threshold is considered >10 μg/mL, this suggests partial resistance in the feline strains we tested (90). This observation is consistent with previous findings reporting MLCs of 1 μg/mL for susceptible strains and up to 100 μg/mL in resistant isolates (20, 91). The suspected resistance in our feline isolates may reflect prior exposure to ronidazole and emphasize the need for diagnostic tools capable of detecting resistant infections to guide appropriate therapy.

In contrast, bovine-origin *T. foetus* strains exhibited markedly higher susceptibility to ronidazole, with an MLC of 1 μg/mL at 24 h and <0.25 μg/mL at 48 h. Alternative nitroimidazoles (metronidazole, tinidazole, and secnidazole) demonstrated similar or improved efficacy. Notably, secnidazole maintained an MLC of 0.5 μg/mL at both time points, highlighting its potential as an alternative treatment. Tinidazole also showed improved efficacy with prolonged treatment. This marks the first study to evaluate ronidazole’s activity against bovine-origin *T. foetus*, pointing to promising avenues for further investigation. However, the lack of comparative data on nitroimidazole efficacy against bovine isolates remains a critical gap.

Our findings on the MLC of nitroimidazoles against *T. foetus* align with existing literature on their efficacy against trichomonas infections. The Centers for Disease Control and Prevention (CDC) notes that metronidazole and tinidazole are the primary treatments for *Trichomonas vaginalis*, with cure rates ranging from 84 to 98% for metronidazole and 92 to 100% for tinidazole. Secnidazole, a newer nitroimidazole, has also demonstrated high efficacy, with some studies reporting cure rates comparable to or exceeding those of metronidazole and tinidazole (92, 93). These data support our observations of secnidazole’s potent activity against bovine-origin *T. foetus* strains and suggest its potential as an effective alternative where other nitroimidazoles are less effective or contraindicated.

The role of propolis as an alternative treatment is particularly compelling in light of regulatory restrictions on nitroimidazoles in food-producing animals. Although there are no direct comparative studies on propolis efficacy against feline- or bovine-origin *T. foetus*, studies in other protozoan infections support its potential. For instance, Brazilian propolis eradicated *T. vaginalis* at 500 μg/mL (83) while Cuban propolis achieved similar effects at much lower concentrations (3.2–9.1 μg/mL) (94). In contrast, Egyptian propolis required concentrations as high as 75,000 μg/mL against *Trichomonas gallinae* (95). Hungarian propolis has demonstrated antiparasitic activity in avian studies at concentrations ranging from 1,100 to 5,000 μg/mL (44, 96, 97). Our data suggest that bovine-origin *T. foetus* is particularly sensitive to propolis, which may be promising for local treatments in breeding bulls and for reducing infection reservoirs in cattle.

Nevertheless, variability in propolis composition remains a major challenge. Standardization of key active compounds — such as flavonoids and phenolic acids — and detailed pharmacokinetic studies are essential to enable reproducible and clinically relevant outcomes. In this study, we observed distinct differences in the sensitivity of feline and bovine *T. foetus* isolates to both propolis and nitroimidazoles. The partial resistance of feline strains to ronidazole highlights the importance of continued surveillance and alternative therapies to combat AMR. Future studies should prioritize the development of reliable diagnostic assays to differentiate treatment failure due to resistance, reinfection, or suboptimal dosing regimens.

The variability in the effective concentrations of propolis observed in our study aligns with the significant differences reported in other *Trichomonas* species. For example, a study from Hungary found that ethanolic tincture of propolis from the Észak-Alföld region had a minimum eradication concentration (MEC) ranging from 2.5 to 5 mg/mL against *T. gallinae* (96). In contrast, an Egyptian study reported that an aqueous propolis tincture required concentrations as high as 50 mg/mL to fully inhibit the growth of *T. gallinae* within 48 h (95). These findings underscore the critical influence of botanical and geographical factors on the composition and efficacy of propolis, particularly in terms of its flavonoid and phenolic content, and highlight the need for standardization to ensure reproducible and reliable antiparasitic effects.

In summary, our findings contribute to the growing body of evidence supporting natural, sustainable alternatives to conventional antimicrobials in veterinary medicine. By highlighting the potential of propolis and identifying strain-dependent differences in susceptibility, this work aligns with the One Health objective of mitigating AMR while safeguarding animal health and productivity.

## 5 Conclusion

This study provides compelling *in vitro* evidence that Hungarian propolis exhibits significant antiparasitic activity against both feline- and bovine-derived *T. foetus* strains, with particularly marked efficacy against bovine isolates. These findings are noteworthy given the regulatory restrictions on nitroimidazole use in food-producing animals and the emerging partial resistance of feline strains to ronidazole, underscoring the pressing need for novel, sustainable alternatives in veterinary practice.

Importantly, our results highlight the potential of propolis as a natural antimicrobial agent that aligns with the One Health concept of integrated approaches to combat AMR while preserving animal health and productivity. However, the inherent variability in propolis composition — driven by geographical, botanical, and extraction factors — necessitates standardization of its active constituents to ensure reproducible efficacy and safety. Furthermore, comprehensive *in vivo* studies, including pharmacokinetic profiling and safety assessments, are essential before propolis can be integrated into therapeutic regimens.

Future research should also prioritize the development of reliable diagnostic tools to detect resistant *T. foetus* strains, enabling targeted interventions and supporting stewardship efforts to mitigate AMR spread. Collectively, this work contributes to the expanding evidence base supporting the use of natural compounds as adjuncts or alternatives to conventional antimicrobials, offering promising avenues for sustainable disease management in veterinary medicine.

## Data Availability

The original contributions presented in the study are included in the article/Supplementary material, further inquiries can be directed to the corresponding author.

## Supplementary material

SUPPLEMENTARY VIDEO 1 Live microscopy of motile Tritrichomonas foetus trophozoites observed under phase-contrast illumination (400×). The video illustrates the active flagellar motion and characteristic tumbling behavior of the parasite.

## References

[1] Thornton PK. Livestock production: recent trends, future prospects. Philos Trans R Soc Lond Ser B Biol Sci. (2010) 365:2853–67. doi: 10.1098/rstb.2010.0134, PMID: 20713389 PMC2935116

[2] Dehove A Commault J Petitclerc M Teissier M Macé J. Economic analysis and costing of animal health: a literature review of methods and importance. Rev Sci Tech. (2012) 31:605–17. doi: 10.20506/rst.31.2.2146, PMID: 23413736

[3] Daszak P Cunningham AA Hyatt AD. Emerging infectious diseases of wildlife-threats to biodiversity and human health. Science. (2000) 287:443–9. doi: 10.1126/science.287.5452.443, PMID: 10642539

[4] Slapeta J Müller N Stack CM Walker G Lew-Tabor A Tachezy J . Comparative analysis of *Tritrichomonas foetus* (Riedmüller, 1928) cat genotype, *T. foetus* (Riedmüller, 1928) cattle genotype and *Tritrichomonas suis* (Davaine, 1875) at 10 DNA loci. Int J Parasitol. (2012) 42:1143–9. doi: 10.1016/j.ijpara.2012.10.004, PMID: 23123273

[5] Slapeta J Craig S McDonell D Emery D. *Tritrichomonas foetus* from domestic cats and cattle are genetically distinct. Exp Parasitol. (2010) 126:209–13. doi: 10.1016/j.exppara.2010.04.024, PMID: 20438726

[6] Sun Z Stack C Šlapeta J. Sequence differences in the diagnostic region of the cysteine protease 8 gene of *Tritrichomonas foetus* parasites of cats and cattle. Vet Parasitol. (2012) 186:445–9. doi: 10.1016/j.vetpar.2011.12.001, PMID: 22204891

[7] Reinmann K Müller N Kuhnert P Campero CM Leitsch D Hess M . *Tritrichomonas foetus* isolates from cats and cattle show minor genetic differences in unrelated loci ITS-2 and EF-1α. Vet Parasitol. (2012) 185:138–44. doi: 10.1016/j.vetpar.2011.09.032, PMID: 22000167

[8] Rivera WL Lupisan AJB Baking JMP. Ultrastructural study of a tetratrichomonad isolated from pig fecal samples. Parasitol Res. (2008) 103:1311–6. doi: 10.1007/s00436-008-1134-x, PMID: 18682985

[9] Miró G Hernández L Montoya A Arranz-Solís D Dado D Rojo-Montejo S . First description of naturally acquired *Tritrichomonas foetus* infection in a Persian cattery in Spain. Parasitol Res. (2011) 109:1151–4. doi: 10.1007/s00436-011-2359-7, PMID: 21509446

[10] Kuehner KA Marks SL Kass PH Sauter-Louis C Grahn RA Barutzki D . *Tritrichomonas foetus* infection in purebred cats in Germany: prevalence of clinical signs and the role of co-infection with other enteroparasites. J Feline Med & Surg. (2011) 13:251–8. doi: 10.1016/j.jfms.2010.12.002, PMID: 21288749 PMC10832821

[11] Bissett SA Stone ML Malik R Norris JM O’Brien C Mansfield CS . Observed occurrence of *Tritrichomonas foetus* and other enteric parasites in Australian cattery and shelter cats. J Feline Med Surg. (2009) 11:803–7. doi: 10.1016/j.jfms.2009.02.001, PMID: 19285895 PMC11135510

[12] Tysnes K Gjerde B Nødtvedt A Skancke E. A cross-sectional study of *Tritrichomonas foetus* infection among healthy cats at shows in Norway. Acta Vet Scand. (2011) 53:39. doi: 10.1186/1751-0147-53-39, PMID: 21689400 PMC3155830

[13] Kingsbury DD Marks SL Cave NJ Grahn RA. Identification of *Tritrichomonas foetus* and *Giardia* spp. infection in pedigree show cats in New Zealand. N Z Vet J. (2010) 58:6–10. doi: 10.1080/00480169.2010.65054, PMID: 20200569

[14] Xenoulis PG Lopinski DJ Read SA Suchodolski JS Steiner JM. Intestinal *Tritrichomonas foetus* infection in cats: a retrospective study of 104 cases. J Feline Med Surg. (2013) 15:1098–103. doi: 10.1177/1098612X13495024, PMID: 23838083 PMC10816472

[15] Yao C Köster LS. *Tritrichomonas foetus* infection, a cause of chronic diarrhea in the domestic cat. Vet Res. (2015) 46:35. doi: 10.1186/s13567-015-0169-0, PMID: 25880025 PMC4364588

[16] Granizo JJ Pía Rodicio M Manso FJ Giménez MJ. Tinidazole: a classical anaerobical drug with multiple potential uses nowadays. Rev Esp Quimioter. (2009) 22:106–14. PMID: 19544102

[17] Fung HB Doan T-L. Tinidazole: a nitroimidazole antiprotozoal agent. Clin Ther. (2005) 27:1859–84. doi: 10.1016/j.clinthera.2005.12.012, PMID: 16507373

[18] Gookin JL Stauffer SH Coccaro MR Poore MF Levy MG Papich MG. Efficacy of tinidazole for treatment of cats experimentally infected with *Tritrichomonas foetus*. Am J Vet Res. (2007) 68:1085–8. doi: 10.2460/ajvr.68.10.1085, PMID: 17916015

[19] LeVine DN Papich MG Gookin JL Davidson GS Davis JL Hayes RB. Ronidazole pharmacokinetics after intravenous and oral immediate-release capsule administration in healthy cats. J Feline Med Surg. (2011) 13:244–50. doi: 10.1016/j.jfms.2010.12.001, PMID: 21239199 PMC10832812

[20] Gookin JL Copple CN Papich MG Poore MF Stauffer SH Birkenheuer AJ . Efficacy of ronidazole for treatment of feline *Tritrichomonas foetus* infection. J Vet Intern Med. (2006) 20:536–43. doi: 10.1111/j.1939-1676.2006.tb02893.x, PMID: 16734086

[21] Samuelson J. Why metronidazole is active against both Bacteria and parasites. Antimicrob Agents Chemother. (1999) 43:1533–41. doi: 10.1128/AAC.43.7.1533, PMID: 10390199 PMC89320

[22] Gookin JL Hanrahan K Levy MG. The conundrum of feline Trichomonosis. J Feline Med Surg. (2017) 19:261–74. doi: 10.1177/1098612X17693499, PMID: 28245739 PMC11119535

[23] Gookin JL Breitschwerdt EB Levy MG Gager RB Benrud JG. Diarrhea associated with trichomonosis in cats. J Am Vet Med Assoc. (1999) 215:1450–4. doi: 10.2460/javma.1999.215.10.1450, PMID: 10579040

[24] Foster DM Gookin JL Poore MF Stebbins ME Levy MG. Outcome of cats with diarrhea and *Tritrichomonas foetus* infection. J Am Vet Med Assoc. (2004) 225:888–92. doi: 10.2460/javma.2004.225.888, PMID: 15485048

[25] Kather EJ Marks SL Kass PH. Determination of the *in vitro* susceptibility of feline tritrichomonas foetus to 5 antimicrobial agents. J Vet Intern Med. (2007) 21:966–70. doi: 10.1111/j.1939-1676.2007.tb03050.x, PMID: 17939550

[26] Yule A Skirrow SZ BonDuran RH. Bovine trichomoniasis. Parasitol Today. (1989) 5:373–7. doi: 10.1016/0169-4758(89)90298-6, PMID: 15463161

[27] Michi AN Favetto PH Kastelic J Cobo ER. A review of sexually transmitted bovine trichomoniasis and campylobacteriosis affecting cattle reproductive health. Theriogenology. (2016) 85:781–91. doi: 10.1016/j.theriogenology.2015.10.037, PMID: 26679515

[28] Parsonson IM Clark BL Dufty JH. Early pathogenesis and pathology of *Tritrichomonas foetus* infection in virgin heifers. J Comp Pathol. (1976) 86:59–66. doi: 10.1016/0021-9975(76)90028-1, PMID: 1254746

[29] Okamoto S Wakui M Kobayashi H Sato N Ishida A Tanabe M . *Trichomonas foetus* meningoencephalitis after allogeneic peripheral blood stem cell transplantation. Bone Marrow Transplant. (1998) 21:89–91. doi: 10.1038/sj.bmt.1701032, PMID: 9486501

[30] Muzny CA Schwebke JR Nyirjesy P Kaufman G Mena LA Lazenby GB . Efficacy and safety of single Oral dosing of Secnidazole for trichomoniasis in women: results of a phase 3, randomized, double-blind, placebo-controlled, delayed-treatment study. Clin Infect Dis. (2021) 73:e1282–9. doi: 10.1093/cid/ciab242, PMID: 33768237 PMC8442793

[31] Muzny CA Van Gerwen OT. Secnidazole for trichomoniasis in women and men. Sex Med Rev. (2022) 10:255–62. doi: 10.1016/j.sxmr.2021.12.004, PMID: 35153156 PMC11019772

[32] Cheung W Russo C Maher S Malik R Šlapeta J. Successful use of secnidazole to manage a giardiosis outbreak in a shelter. Vet Parasitol. (2019) 274:108911. doi: 10.1016/j.vetpar.2019.08.005, PMID: 31499401

[33] Da Silva AS Castro VSP Tonin AA Brendler S Costa MM Jaques JA . Secnidazole for the treatment of giardiasis in naturally infected cats. Parasitol Int. (2011) 60:429–32. doi: 10.1016/j.parint.2011.06.024, PMID: 21763779

[34] Meites E Gaydos CA Hobbs MM Kissinger P Nyirjesy P Schwebke JR . A review of evidence-based Care of Symptomatic Trichomoniasis and Asymptomatic *Trichomonas vaginalis* infections. Clin Infect Dis. (2015) 61:S837–48. doi: 10.1093/cid/civ738, PMID: 26602621 PMC4657597

[35] Kissinger PJ Gaydos CA Seña AC Scott McClelland R Soper D Secor WE . Diagnosis and management of *Trichomonas vaginalis*: summary of evidence reviewed for the 2021 centers for disease control and prevention sexually transmitted infections treatment guidelines. Clin Infect Dis. (2022) 74:S152–61. doi: 10.1093/cid/ciac030, PMID: 35416973 PMC9006969

[36] Workowski KA. Centers for Disease Control and Prevention sexually transmitted diseases treatment guidelines. Clin Infect Dis. (2015) 61:S759–62. doi: 10.1093/cid/civ771 26602614

[37] Tabari MA Poźniak B Abrishami A Moradpour AA Shahavi MH Kazemi S . Antitrichomonal activity of metronidazole-loaded lactoferrin nanoparticles in pigeon trichomoniasis. Parasitol Res. (2021) 120:3263–72. doi: 10.1007/s00436-021-07263-z, PMID: 34342682

[38] Belen Rivero M Emilio Luque M Eugenia Abdala M Elias Luna B Di Lullo D Eduardo Echaide I . *In vitro* susceptibility to metronidazole of *Tritrichomonas foetus* bovine isolates from Argentina. Acta Parasitol. (2019) 64:232–5. doi: 10.2478/s11686-019-00031-1, PMID: 30783992

[39] Tuska-Szalay B Jerzsele Á Hornok S. Antiprotozoal agents used in veterinary medicine. Magy Allatorv Lapja. (2024) 146:487–500. doi: 10.56385/magyallorv.2024.08.487-500

[40] Love D Fajt VR Hairgrove T Jones M Thompson JA. Metronidazole for the treatment of *Tritrichomonas foetus* in bulls. BMC Vet Res. (2017) 13:107. doi: 10.1186/s12917-017-0999-2, PMID: 28410582 PMC5391598

[41] de Koning HP. Drug resistance in protozoan parasites. Emerg Top Life Sci. (2017) 1:627–32. doi: 10.1042/ETLS20170113, PMID: 33525852 PMC7289004

[42] Kovács L Nagy D Könyves L Jerzsele Á Kerek Á. Antimicrobial properties of essential oils – animal health aspects. Magy Allatorv Lapja. (2023) 145:497–510. doi: 10.56385/magyallorv.2023.08.497-510

[43] Sebők C Márton RA Meckei M Neogrády Z Mátis G. Antimicrobial peptides as new tools to combat infectious diseases. Magy Allatorv Lapja. (2024) 146:181–91. doi: 10.56385/magyallorv.2024.03.181-191

[44] Kerek Á Csanády P Jerzsele Á. Antiprotozoal and antifungal efficiency of propolis – part 2. Magy Allatorv Lapja. (2022) 144:691–704.

[45] Almuhayawi MS. Propolis as a novel antibacterial agent. Saudi J Biol Sci. (2020) 27:3079–86. doi: 10.1016/j.sjbs.2020.09.016, PMID: 33100868 PMC7569119

[46] Santos LM Fonseca MS Sokolonski AR Deegan KR Araujo RPC Umsza-Guez MA . Propolis: types, composition, biological activities, and veterinary product patent prospecting. J Sci Food Agric. (2020) 100:1369–82. doi: 10.1002/jsfa.10024, PMID: 31487405

[47] Przybyłek I Karpiński TM. Antibacterial properties of Propolis. Molecules. (2019) 24:2047. doi: 10.3390/molecules24112047, PMID: 31146392 PMC6600457

[48] Kowacz M Pollack GH. Propolis-induced exclusion of colloids: possible new mechanism of biological action. Colloid Interface Sci Commun. (2020) 38:100307. doi: 10.1016/j.colcom.2020.100307, PMID: 32864353 PMC7442903

[49] Rivera-Yañez N Rivera-Yañez CR Pozo-Molina G Méndez-Catalá CF Reyes-Reali J Mendoza-Ramos MI . Effects of Propolis on infectious diseases of medical relevance. Biology (Basel). (2021) 10:428. doi: 10.3390/biology10050428, PMID: 34065939 PMC8151468

[50] Silva-Carvalho R Baltazar F Almeida-Aguiar C. Propolis: a complex natural product with a plethora of biological activities that can be explored for drug development. Evid Based Complement Alternat Med. (2015) 2015:e206439. doi: 10.1155/2015/206439, PMID: 26106433 PMC4461776

[51] Regueira-Neto M d S Tintino SR Rolón M Coronal C Vega MC de Queiroz Balbino V . Antitrypanosomal, antileishmanial and cytotoxic activities of Brazilian red propolis and plant resin of *Dalbergia ecastaphyllum* (L) Taub. Food Chem Toxicol. (2018) 119:215–21. doi: 10.1016/j.fct.2018.04.029, PMID: 29665415

[52] Gómez-Caravaca AM Gómez-Romero M Arráez-Román D Segura-Carretero A Fernández-Gutiérrez A. Advances in the analysis of phenolic compounds in products derived from bees. J Pharm Biomed Anal. (2006) 41:1220–34. doi: 10.1016/j.jpba.2006.03.002, PMID: 16621403

[53] Farnesi AP Aquino-Ferreira R De Jong D Bastos JK Soares AEE. Effects of stingless bee and honey bee propolis on four species of bacteria. Genet Mol Res. (2009) 8:635–40. doi: 10.4238/vol8-2kerr023, PMID: 19554760

[54] Fathi Hafshejani S Lotfi S Rezvannejad E Mortazavi M Riahi-Madvar A. Correlation between total phenolic and flavonoid contents and biological activities of 12 ethanolic extracts of Iranian propolis. Food Sci Nutr. (2023) 11:4308–25. doi: 10.1002/fsn3.3356, PMID: 37457164 PMC10345684

[55] Asem N Abdul Gapar NA Abd Hapit NH Omar EA. Correlation between total phenolic and flavonoid contents with antioxidant activity of Malaysian stingless bee propolis extract. J Apic Res. (2020) 59:437–42. doi: 10.1080/00218839.2019.1684050

[56] Abduh MY Shafitri TR Elfahmi E. Chemical profiling, bioactive compounds, antioxidant, and anti-inflammatory activities of Indonesian propolis extract produced by *Tetragonula laeviceps*. Heliyon. (2024) 10:e38736. doi: 10.1016/j.heliyon.2024.e38736, PMID: 39397935 PMC11471232

[57] Siheri W Ebiloma GU Igoli JO Gray AI Biddau M Akrachalanont P . Isolation of a novel flavanonol and an Alkylresorcinol with highly potent anti-Trypanosomal activity from Libyan Propolis. Molecules. (2019) 24:1041. doi: 10.3390/molecules24061041, PMID: 30884752 PMC6471328

[58] Pomothy JM Barna RF Gere E. The effects of the rosmarinic acid in livestock animals: literature review. Magy Allatorv Lapja. (2020) 142:567–76.

[59] Antwi CA Amisigo CM Adjimani JP Gwira TM. *In vitro* activity and mode of action of phenolic compounds on *Leishmania donovani*. PLoS Negl Trop Dis. (2019) 13:e0007206. doi: 10.1371/journal.pntd.0007206, PMID: 30802252 PMC6405172

[60] Fonseca-Silva F Canto-Cavalheiro MM Menna-Barreto RFS Almeida-Amaral EE. Effect of apigenin on *Leishmania amazonensis* is associated with reactive oxygen species production followed by mitochondrial dysfunction. J Nat Prod. (2015) 78:880–4. doi: 10.1021/acs.jnatprod.5b00011, PMID: 25768915

[61] Mallo N Lamas J Leiro JM. Hydrogenosome metabolism is the key target for antiparasitic activity of resveratrol against *Trichomonas vaginalis*. Antimicrob Agents Chemother. (2013) 57:2476–84. doi: 10.1128/AAC.00009-13, PMID: 23478970 PMC3716124

[62] Bolaños V Díaz-Martínez A Soto J Marchat LA Sanchez-Monroy V Ramírez-Moreno E. Kaempferol inhibits *Entamoeba histolytica* growth by altering cytoskeletal functions. Mol Biochem Parasitol. (2015) 204:16–25. doi: 10.1016/j.molbiopara.2015.11.004, PMID: 26620675

[63] Sen G Mukhopadhyay S Ray M Biswas T. Quercetin interferes with iron metabolism in *Leishmania donovani* and targets ribonucleotide reductase to exert leishmanicidal activity. J Antimicrob Chemother. (2008) 61:1066–75. doi: 10.1093/jac/dkn053, PMID: 18285311

[64] Bortoleti BT d S Tomiotto-Pellissier F Gonçalves MD Miranda-Sapla MM Assolini JP Carloto AC . Caffeic acid has antipromastigote activity by apoptosis-like process; and anti-amastigote by TNF-α/ROS/NO production and decreased of iron availability. Phytomedicine. (2019) 57:262–70. doi: 10.1016/j.phymed.2018.12.035, PMID: 30802712

[65] Huang S Zhang C-P Wang K Li GQ Hu F-L. Recent advances in the chemical composition of Propolis. Molecules. (2014) 19:19610–32. doi: 10.3390/molecules191219610, PMID: 25432012 PMC6271758

[66] Teles CBG Moreira-Dill LS Silva A d A Facundo VA de Azevedo WF da Silva LHP . A Lupane-triterpene isolated from *Combretum leprosum* Mart. Fruit extracts that interferes with the intracellular development of *Leishmania (L.) amazonensis in vitro*. BMC Complement Altern Med. (2015) 15:165. doi: 10.1186/s12906-015-0681-9 26048712 PMC4457080

[67] De Pablos LM González G Rodrigues R García Granados A Parra A Osuna A. Action of a pentacyclic triterpenoid, maslinic acid, against *Toxoplasma gondii*. J Nat Prod. (2010) 73:831–4. doi: 10.1021/np900749b, PMID: 20441162

[68] Maróstica Junior MR Daugsch A Moraes CS Queiroga CL Pastore GM Parki YK. Comparison of volatile and polyphenolic compounds in Brazilian green propolis and its botanical origin *Baccharis dracunculifolia*. Cienc Tecnol Aliment. (2008) 28:178–81. doi: 10.1590/S0101-20612008000100026

[69] Cunha S Sawaya ACHF Caetano FM Shimizu MT Marcucci MC Drezza FT . Factors that influence the yield and composition of Brazilian propolis extracts. J Braz Chem Soc. (2004) 15:964–70. doi: 10.1590/S0103-50532004000600026

[70] Galeotti F Maccari F Fachini A Volpi N. Chemical composition and antioxidant activity of Propolis prepared in different forms and in different solvents useful for finished products. Foods. (2018) 7:41. doi: 10.3390/foods7030041, PMID: 29562665 PMC5867556

[71] Campero CM. Use of DMSO for the cryopreservation of *Tritrichomonas foetus* in liquid nitrogen. Vet Parasitol. (1989) 31:339–43. doi: 10.1016/0304-4017(89)90083-6, PMID: 2763452

[72] Moreira L Dias LG Pereira JA Estevinho L. Antioxidant properties, total phenols and pollen analysis of propolis samples from Portugal. Food Chem Toxicol. (2008) 46:3482–5. doi: 10.1016/j.fct.2008.08.025, PMID: 18804144

[73] Dias LG Pereira AP Estevinho LM. Comparative study of different Portuguese samples of propolis: pollinic, sensorial, physicochemical, microbiological characterization and antibacterial activity. Food Chem Toxicol. (2012) 50:4246–53. doi: 10.1016/j.fct.2012.08.056, PMID: 22981908

[74] Bankova V Popova M Trusheva B. The phytochemistry of the honeybee. Phytochemistry. (2018) 155:1–11. doi: 10.1016/j.phytochem.2018.07.007, PMID: 30053651

[75] Silici S Kutluca S. Chemical composition and antibacterial activity of propolis collected by three different races of honeybees in the same region. J Ethnopharmacol. (2005) 99:69–73. doi: 10.1016/j.jep.2005.01.046, PMID: 15848022

[76] Popova M Giannopoulou E Skalicka-Woźniak K Graikou K Widelski J Bankova V . Characterization and biological evaluation of Propolis from Poland. Molecules. (2017) 22:1159. doi: 10.3390/molecules22071159, PMID: 28696397 PMC6152113

[77] Bankova V Popova M Trusheva B. Propolis volatile compounds: chemical diversity and biological activity: a review. Chem Cent J. (2014) 8:28. doi: 10.1186/1752-153X-8-28, PMID: 24812573 PMC4014088

[78] Meingassner JG Thurner J. Strain of *Trichomonas vaginalis* resistant to metronidazole and other 5-nitroimidazoles. Antimicrob Agents Chemother. (1979) 15:254–7. doi: 10.1128/AAC.15.2.254, PMID: 311617 PMC352642

[79] Meingassner JG Mieth H Czok R Lindmark DG Müller M. Assay conditions and the demonstration of nitroimidazole resistance in *Tritrichomonas foetus*. Antimicrob Agents Chemother. (1978) 13:1–3. doi: 10.1128/AAC.13.1.1, PMID: 626482 PMC352174

[80] Ghosh AP Aycock C Schwebke JR. *In vitro* study of the susceptibility of clinical isolates of *Trichomonas vaginalis* to metronidazole and secnidazole. Antimicrob Agents Chemother. (2018) 62:e02329–17. doi: 10.1128/AAC.02329-17, PMID: 29439963 PMC5913958

[81] Hezarjaribi HZ Mollarostami F Ebrahimnejad P Esboei BR Fakhar M Sadeghi-Ghadi Z. Promising potent *in vitro* activity of curcumin and quercetin nano-niosomes against *Trichomonas vaginalis*. Ann Parasitol. (2022) 68:263–73. doi: 10.17420/ap6802.432, PMID: 35809560

[82] Rivero MB Luque ME Abdala ME Luna BE Di Lullo D Carranza PG . Flow cytometry evaluation of *in vitro* susceptibility of bovine isolates of *Tritrichomonas foetus* to metronidazole. Vet Parasitol. (2019) 267:84–9. doi: 10.1016/j.vetpar.2019.02.004, PMID: 30878091

[83] Sena-Lopes Â Bezerra FSB das Neves RN de Pinho RB Silva MT d O Savegnago L . Chemical composition, immunostimulatory, cytotoxic and antiparasitic activities of the essential oil from Brazilian red propolis. PLoS One. (2018) 13:e0191797. doi: 10.1371/journal.pone.0191797, PMID: 29390009 PMC5794096

[84] Yazdani N Youssefi MR Tabari MA. Antitrichomonal activity of nanoemulsion of carvacrol on *Trichomonas galline*: formulation development and *in vitro* characterization. Ann Parasitol. (2022) 68:151–7. doi: 10.17420/ap6801.419, PMID: 35491905

[85] Clinical and Laboratory Standards Institute. Methods for dilution antimicrobial susceptibility tests for Bacteria that grow aerobically. 11. th ed. Wayne, PA: Clinical and Laboratory Standards Institute (2018).

[86] McKight PE Najab J. Kruskal-Wallis Test In: Editors Weiner IB Craighead WE. The Corsini Encyclopedia of psychology. Hoboken, NJ: John Wiley & Sons, Ltd (2010). 1.

[87] R Core Team. (2020). R: A language and environment for statistical computing. R Foundation for Statistical Computing, Vienna, Austria. Available online at: http://www.r-project.org/index.html [Accessed September 4, 2025].

[88] Freitas SF Shinohara L Sforcin JM Guimarães S. *In vitro* effects of propolis on *Giardia duodenalis* trophozoites. Phytomedicine. (2006) 13:170–5. doi: 10.1016/j.phymed.2004.07.008, PMID: 16428024

[89] Pontin K Da Silva Filho AA Santos FF Silva MLAE Cunha WR Nanayakkara NPD . *In vitro* and *in vivo* antileishmanial activities of a Brazilian green propolis extract. Parasitol Res. (2008) 103:487–92. doi: 10.1007/s00436-008-0970-z, PMID: 18491139

[90] Rush GM Šlapeta J. Evidence of self-resolution of feline trichomonosis in a pair of single household cats due to ronidazole-resistant *Tritrichomonas foetus*. Vet Parasitol. (2021) 300:109609. doi: 10.1016/j.vetpar.2021.109609, PMID: 34735847

[91] Gookin JL Stauffer SH Dybas D Cannon DH. Documentation of *in vivo* and *in vitro* aerobic resistance of feline *Tritrichomonas foetus* isolates to Ronidazole. J Vet Intern Med. (2010) 24:1003–7. doi: 10.1111/j.1939-1676.2010.0534.x, PMID: 20492492

[92] Trichomoniasis (2022). STI treatment guidelines. Available online at: https://www.cdc.gov/std/treatment-guidelines/trichomoniasis.htm [Accessed May 24, 2025]

[93] Darvin SS. (2025). Trichomoniasis Treatment & Management: Approach considerations, pharmacologic therapy, diet and activity. Available online at: https://emedicine.medscape.com/article/230617-treatment [Accessed May 24, 2025]

[94] Monzote Fidalgo L Sariego Ramos I García Parra M Cuesta-Rubio O Márquez Hernández I Campo Fernández M . Activity of Cuban propolis extracts on *leishmania amazonensis* and *Trichomonas vaginalis*. Nat Prod Commun. (2011) 6:973–6. doi: 10.1177/1934578X1100600712, PMID: 21834236

[95] Arafa MI Hassan HHK Magmoed WGM Abdel-Rahman MF. Study the effect of aqueous extract of propolis on *Trichomonas gallinae*, *in vitro*. Assiut Vet Med J. (2016) 62:82–8. doi: 10.21608/avmj.2016.169993

[96] Kerek Á Csanády P Tuska-Szalay B Kovács L Jerzsele Á. *In vitro* efficacy of Hungarian propolis against bacteria, yeast, and *Trichomonas gallinae* isolated from pigeons—a possible antibiotic alternative? Resources. (2023) 12:101. doi: 10.3390/resources12090101

[97] Kerek Á Csanády P Jerzsele Á. Antibacterial efficiency of propolis – part 1. Magy Allatorv Lapja. (2022) 144:285–98.

