# Supplementary material for: Antimicrobial resistance and natural alternatives: in vitro efficacy of Hungarian propolis against feline and bovine Tritrichomonas foetus
Source: Front Vet Sci. 2025 Nov 14;12:1635358. doi: 10.3389/fvets.2025.1635358 (PMC12662178; doi:10.3389/fvets.2025.1635358)
Supplement: Supplementary file 1 [file Table_1.docx]

**Supplementary Table 1** Statistical groupwise comparisons of parasite strains were conducted based on species, treatment time, concentration, and treatment materials using the Kruskal-Wallis test. The analyses were performed regarding trophozoite count, and corresponding p-values are presented in the table.

|  | Strain | Length of treatment | Concentration | Treatment |
| --- | --- | --- | --- | --- |
| Feline (all) | 0.9939 | 0.0144* | <0.0001* | 0.0225* |
| Propolis | 0.9826 | 0.0099* | <0.0001* |  |
| Ronidazole | 0.9857 | 0.6146 | <0.0001* |  |
| Bovine (all) | 0.9943 | 0.0317* | <0.0001* | <0.0001* |
| Propolis | 0.9700 | 0.1393 | <0.0001* |  |
| Ronidazole | 0.9988 | 0.0112* | <0.0001* |  |
| Metronidazole | 0.9943 | 0.0317* | <0.0001* |  |
| Tinidazole | 0.9973 | 0.0941 | <0.0001* |  |
| Secnidazole | 0.9983 | 0.9158 | <0.0001* |  |

* significant difference
